# Effects of land use type, spatial patterns and host presence on *Leishmania tropica* vectors activity

**DOI:** 10.1186/s13071-019-3562-0

**Published:** 2019-06-25

**Authors:** Yoni Waitz, Shlomit Paz, David Meir, Dan Malkinson

**Affiliations:** 1 Department of Geography and Environmental Studies, University of Haifa, Haifa, Israel; 2 Mitzpe Abirim, Israel; 3 Shamir Research Institute, University of Haifa, Haifa, Israel

**Keywords:** Leishmaniasis, *Phlebotomus sergenti*, *Phlebotomus arabicus*, Rock hyrax, Land-use effect

## Abstract

**Background:**

Leishmaniasis is a vector-borne disease, caused by the infection of *Leishmania* parasites which are transmitted by the bite of infected female phlebotomine sand flies. *Leishmania tropica* is transmitted by *Phlebotomus sergenti* and *Phlebotomus arabicus* while the main reservoir host is the rock hyrax. A marked increase in the incidence of cutaneous leishmaniasis (CL) caused by *L. tropica* has been detected in recent years in Israel; it is associated with infections which have emerged in new urban and rural foci. The objective of this study is to contribute to a better understanding of the preferred habitat, spatial activities and host-sand fly relationships of both species of vectors within various types of land use.

**Methods:**

Using CDC-type traps, we investigated the activity levels of sand flies. A field survey was conducted in 2016 at Elifelet, an agricultural village characterized by various types of land use. Movement patterns of *P. sergenti* between rock-piles were investigated by using colour-marked sugar baits and analyses of recapture patterns. In 2017, a survey was conducted in the hilly Jordan River area, by comparing sand flies and rock hyrax activities in relation to the size of rock-piles and vegetation cover.

**Results:**

Both sexes of both species were found to have a clear preference for rocky habitats over other land use types in rural landscapes. Movement patterns of *P. sergenti* were characterized by their high presence close to the rocks and an exponential decrease in their recapture, commensurate with the distance from the rocks. Host-sand fly relationships were found to have a higher correlation between rock hyrax activity levels for females than for males of both species of sand flies. Males exhibited the strongest association with the size of rock-piles.

**Conclusions:**

The results suggest a strong affinity of both phlebotomine vector species to the rocky habitats of the Mediterranean areas. We suggest that rock-piles are associated with populations of rock hyraxes attracting female sand flies seeking blood sources. Rapid human population growth, coupled with intensive land-use changes and the creation of artificial rock-piles, which created potential habitats for both vectors and hosts in the proximity of many settlements, have increased the prevalence of *L. tropica* among the human population in the region.

**Electronic supplementary material:**

The online version of this article (10.1186/s13071-019-3562-0) contains supplementary material, which is available to authorized users.

## Background

The manifestation of zoonotic diseases requires the presence of humans, animal hosts, and vectors capable of transmitting them. Thus, any environmental changes which alter the landscape structure, inhabited by hosts and vectors, may modify diseases occurring in humans, positively or negatively. Along with climate change, anthropogenic modifications of the landscape are continuously taking place, together with the growth of the human population, potentially changing the availability of habitats for hosts and disease vectors. The emergence of zoonotic diseases such as malaria, leishmaniasis and giardiasis, has been shown to increase as a result of changes in land use [1]. In the case of certain diseases, among them leishmaniasis, knowledge gaps exist with respect to the ecology and behaviour of the disease vectors in different land use types.

Leishmaniasis is a vector-borne disease, caused by infection of *Leishmania* parasites which are transmitted by the bite of infected female Phlebotomine sand flies. Every year, 0.7–1.2 million new cases of cutaneous leishmaniasis (CL) occur in more than 85 endemic countries of which about one third are in the Mediterranean region [2]. In the eastern Mediterranean basin, both *Leishmania major* and *Leishmania tropica* (the main parasites in Israel) cause cutaneous leishmaniasis (CL) which is manifested as skin sores, while *Leishmania infantum* causes visceral leishmaniasis (VL) which affects various internal organs, usually the spleen, liver and bone marrow [3].

In Israel, *P. sergenti* and *P. arabicus* were found to be naturally infected and have been shown to be the vectors of *L. tropica*. While *P. sergenti* was found in many studies around the Mediterranean basin as the main vector of *L. tropica*, *P. arabicus* was detected and shown to be a vector only in the Galilee region of Israel [4, 5]. Recent findings indicated that areas affected by *L. tropica* are expanding [3, 6, 7]. One suggested explanation for the increase in human leishmaniasis cases in Israel is the expansion of rock hyrax (*Procavia capensis*) populations, the reservoir of *L. tropica* in the region. A study in northern Israel indicated that 10% of the hyraxes sampled in a rural CL focus in an area to the north of the Sea of Galilee were positive for *L. tropica* [8]. Laboratory and field studies indicate that sand fly vectors of *L. tropica* readily bite hyraxes, infecting them with the parasites [9, 10].

In Israel, the development of new residential neighbourhoods, particularly in the Galilee, the central mountains and the Judean desert near Jerusalem, results in dramatic changes in land use patterns. This process is associated with an increased abundance of artificial rock-piles, a by-product of land preparation for agriculture and construction development. These rock-piles, approximately 2–20 metres in diameter, provide ideal refuge sites for the rock hyrax in close proximity to human habitation and agricultural areas [11]. It has been shown that a higher prevalence of *L*. *tropica* in humans is linked to the expansion of hyrax populations in peri-urban areas [12]. However, only a few studies have investigated the effects of land use on the activity of vectors of *L. tropica* in natural and disturbed habitats and its association with the habitats of hyraxes, such as rock-piles.

Habitat characteristics such as land cover and land-use were demonstrated to be important factors dictating vectors’ activities [13, 14]. Furthermore, soil properties such as moisture and organic matter from agriculture, gardening and waste, may enhance conditions for both vectors and hosts [15, 16]. The role of land use in dictating the sand fly activity patterns was investigated mostly on a regional or macro-scale basis, with an emphasis on the distribution range of the species [17–20]. Several studies have addressed the effects of specific breeding locations, resting and foraging sites and the proximity of farming lands on the sand fly activity [21–25], indicating that facilities for farm animals and agricultural fields are preferred habitats for the phlebotomine species. In addition, other studies demonstrated the influence of changes in land use, such as forest degradation, on the abundance of sand flies [26–28].

The habitats of *L*. *tropica* vectors in hilly and mountainous sites along the eastern Mediterranean zone were explored in several studies which showed the preference of *P. sergenti* for natural rocky habitats such as rock crevices and caves, [29–31] and for artificial rocky habitats such as rock-piles, which provide habitat for rock hyraxes [12, 16, 32]. A preference was also found for dry and wind-protected habitats [16, 30, 32, 33]. Fewer studies dealt with *P. arabicus*, due to its low occurrence and small populations. A single study indicated that *P. arabicus* showed a preference for irrigated habitats [16].

Apart from the macro-scale spatial activity patterns, temporal patterns on a daily scale have also been investigated. Nocturnal activity patterns of *P. sergenti* have been described in several studies, in Israel and elsewhere, as having a higher activity during the hours immediately after dusk; this differs from other species with longer nocturnal activity times [16, 34, 35]. Ecological studies have demonstrated the role of spatial and temporal variability on the sand fliesʼ presence or activity patterns in various habitats. Although the dispersal distances of *Phlebotomus* spp. are considered to be limited to a few hundred metres, some species exhibit higher mobility, up to 2 km; these include *P. ariasi,* particularly the females of this species [36] and *P. papatasi* [37]. Differences in movement patterns and distances have been observed between the sexes. Such differences may be attributed to the presence of Lek communal sites, where males display courtship behaviour [38]. Males tend to aggregate in the hosts’ habitats, whether this are wildlife [39] or human environments [40], defending a discrete territory close to the blood source. So far, no similar data have been presented for the vectors of *L. tropica.*

Regarding the above studies, to the best of our knowledge, no research has specifically assessed the activity patterns of *P. sergenti* or *P. arabicus* in relation to the spatial aspects of different land use categories and host preferences in the rural endemic areas of the Mediterranean region. We hypothesize that the sand fly activity and dispersal patterns will show prominent spatial relationships with the habitats of the hosts (rock hyraxes) and will be more abundant around rock-piles than other land use types.

In this study, we specifically aimed to determine whether the activity and spatial distribution patterns of both vectors of *L. tropica* are associated with particular types of land use in a rural Mediterranean landscape, and whether the patterns differ between the vectors. We hypothesize that sand fly activity patterns are not homogeneous in space: they would be more abundant in locations where the hosts’ presence is more prevalent and movement patterns would reflect meal foraging patterns. In that same landscape, we aimed to assess the extent of the host-sand fly relationships, particularly between the presence of the rock hyraxes and the sand fly activity. Thus, we hypothesized that a positive correlation between the abundance of sand flies and rock hyraxes would be observed.

## Methods

### Study area

**Fig. 1:**
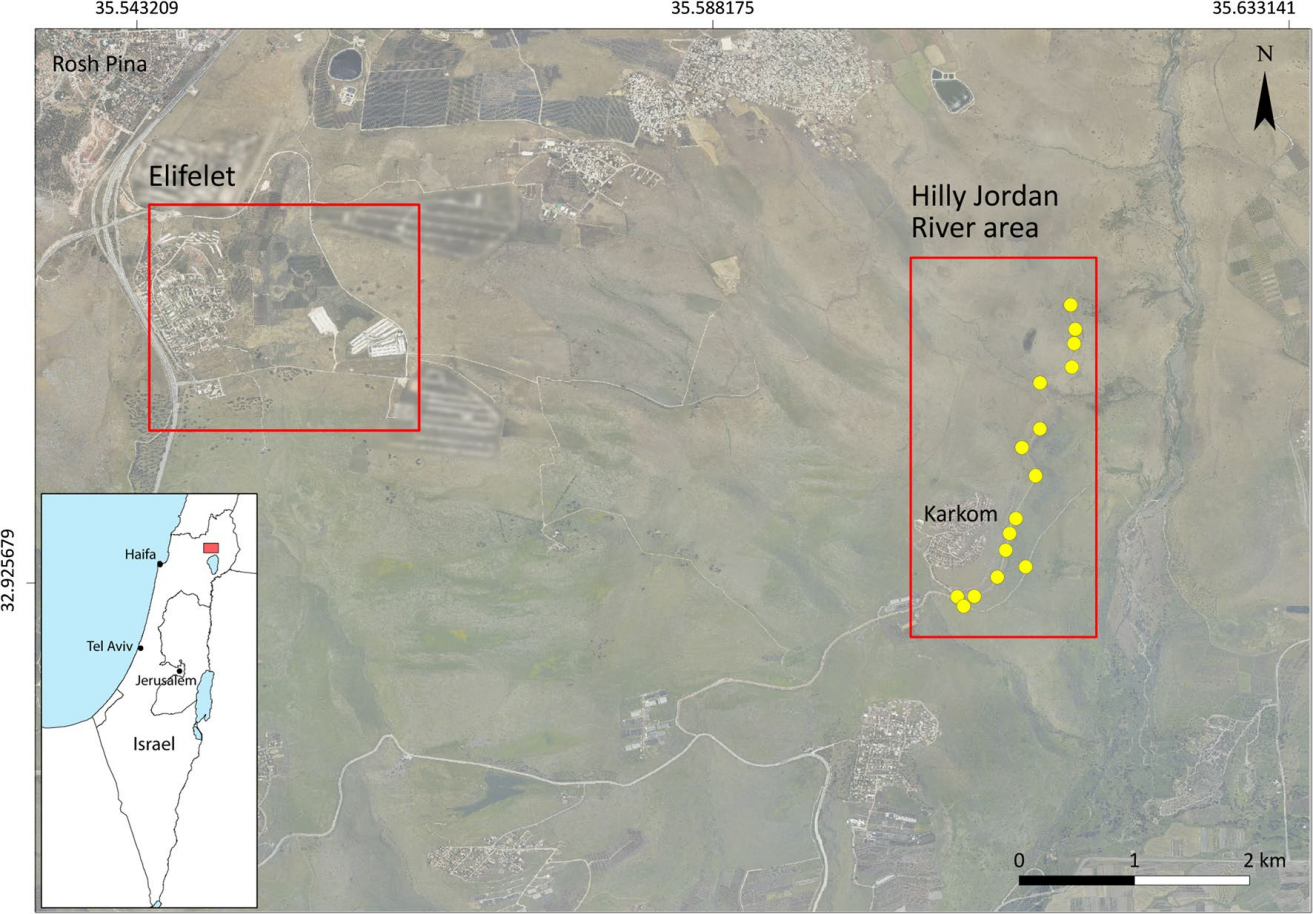
Study area: Korazim Heights and Hilly Jordan River area. Observation points for rock hyraxes and sand fly traps (Aerial photo: Survey of Israel, 2014)

Surveys of the sand flies were conducted in two sites: Elifelet, and the hilly part of the Jordan river area, located in the Korazim Heights, north of the Sea of Galilee. This region has been a hyperendemic zone for *L. tropica* since the late 1990s. It is characterized by basalt rocks and hilly south-east facing topography (see Fig. 1).

### Land use and activity patterns: Elifelet area

#### Site description

Elifelet is an agricultural village, established in the 1950s, 330 metres above sea level, with an average annual precipitation of 500 mm, and average summer temperatures of 26–29 °C, Israel Meteorological Service, 2017. On the eastern side of the village there is a mosaic of agricultural areas and open fields, covered mostly by perennial herbaceous vegetation, often used for cattle grazing. Rock-piles and terraces, mostly basalt, separate the groves (mostly *Prunus* spp. and *Olea europaea*) from the grazing areas.

#### Land use

**Fig. 2:**
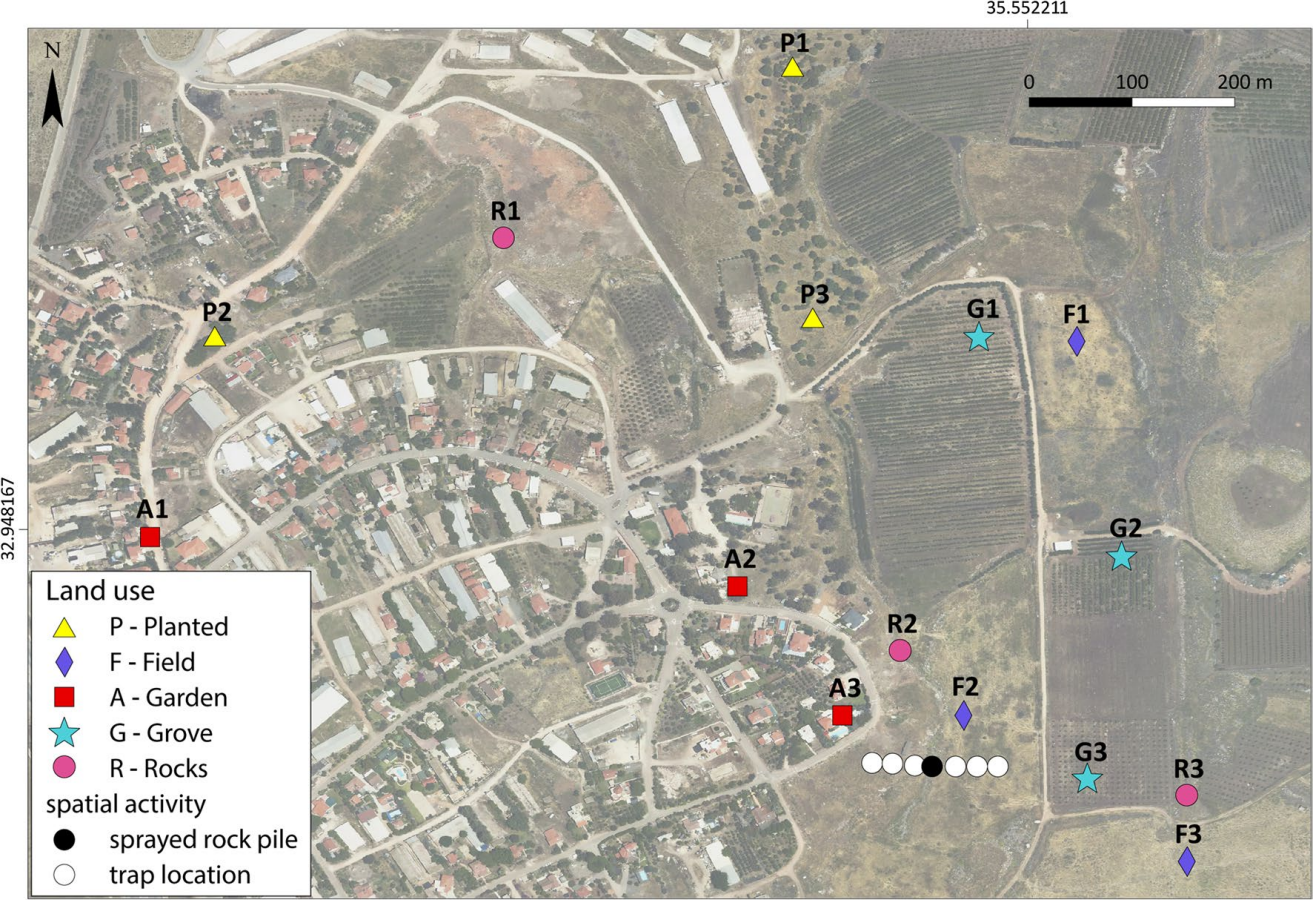
Land use categories in Elifelet. Spatial activity within different types of land-use: trap locations in the open field (Aerial photo: Survey of Israel, 2014)

Open spaces near Elifelet and in the surrounding fields were divided into five land cover categories. Three trapping locations were selected within each category, at least 60 m apart (see Fig. 2): (i) fields: perennial grasslands, used for cattle grazing; (ii) planted: a maquis-like *Ceratonia siliqua* stands, with no irrigation, and used for cattle grazing; (iii) gardens: irrigated gardens in the vicinity of residential or public areas within the village of Elifelet; (iv) groves: planted with prunes and olives, irrigated; and (v) rocks: rock-piles, known to be populated by rock hyraxes.

A survey of sand flies was conducted during the summer of 2016 on three occasions (11th July, 31st July and 24th August). Carbon dioxide (CO_2_) traps (CDC-type) were used for catching sand flies. Each trap contained 1.5 kg of dry ice (frozen CO_2_) as bait.

The traps were set approximately one hour before sunset, and collected half an hour after sunrise, which are the initial and termination activity times of the sand flies [35]. Following the collection, *Phlebotomus* spp. or *Sergentomyia* sand flies were preserved in 70% ethanol for subsequent detailed identification. Other insects were removed. The sand flies, *Phlebotomus* spp. or *Sergentomyia* spp., collected from each trap were identified by sex.

In traps where the number of males or females exceeded 100 individuals, at least 40% were randomly subsampled for identification. These individuals were dissected in Hoyerʼs medium on microscope slides for taxonomical identification. Species were identified based on the morphology of the pharynx, the external genitalia of males and the spermatheca of females, using taxonomic keys [41, 42]. The individuals were divided into three groups: (i) *P. sergenti*; (ii) *P. arabicus*; and (iii) others (e.g. *P. tobbi*, *P. perfiliewi*, *P. simichi*, *Sergentomyia* spp. and others). The last group was not included in further analysis. Capture data were used to assess population activity levels of each species and sex at each location using the N-mixture analysis approach [35].

The traps at each land use type were considered for the repeated observations during the seasonal peak of abundance in the area (see Waitz et al. [35]). Activity analyses were conducted using the *Unmarked* package in R [43]. Eight possible models were tested to estimate the corrected activity from the samplings: (i) a null model with no land use or trap effect; (ii) trap effect; (iii) land use effect; and (iv) trap and land-use effect. Results of the N-mixture analysis indicate that each of the four models was analyzed under a (i) Poisson or a (ii) negative binomial distribution as detailed by Waitz et al. [35].

#### Spatial activity within different types of land-use

Movement patterns of *P. sergenti* among the rock-piles (used as dens for rock hyraxes), and their surroundings during a single night were studied outside Elifelet in an open field, which is used during the spring for cattle grazing. We used a colour-marked sugar bait (developed by Schlein [44]), which enabled us to detect the movement of sand flies from the location of the bait to other trapping locations. Four litres of a solution of 10% sucrose and 5 g/l food dye were sprayed in a circle with a ten-metre diameter on vegetation and on rock surfaces in the centre of a rock-pile outside of Elifelet. The coloured bait was sprayed during the afternoon hours, prior to the setting of the sand fly traps. (see Fig. 2, sprayed rock-piles located in the black point and Additional file 1: Figure S1 for a field viewpoint of the locations of the traps).

CDC-like traps (see trapping methods above) for the sand flies were placed perpendicular to the sprayed rock-pile and oriented toward the nearest house on the south-east side of the village. Six traps were located from the dyed bait source outward: three eastward (away from the houses) and three westward (towards the houses). The traps were placed at an equal distance of 20 m between them (Fig. 2). This experiment was repeated five times during the summer of 2016 (30th August, 6th September, 11th September, 20th September and 5th October), at the same site. Although the coloured baits tended to disappear from the sand flies’ guts after a few days, specific colours (blue, green, red) were used during each sampling session to ensure that the bait eaten could be associated with the specific night of trapping.

Marked sand flies were separated from the non-marked sand flies. Both the marked and the non-marked sand flies were counted, dissected and identified for all traps. Coloured *P. sergenti* were counted for each trap. Linear regressions and comparisons of linear and non-linear models were used to assess the relationship between the distance and the direction on the sand flies’ movements. Four models were tested: (i) Distance only (linear model); (ii) Distance + Direction (linear model); (iii) Distance (exponential decrease model); and (iv) Distance + Direction (exponential decrease model).

### Host-sand fly relationships: the hilly area of the Jordan River

The study site is located along a dirt road in a landscape of rolling hills in the vicinity of Karkom, a small agricultural village (Fig. 1), characterized by abundant rocky habitats favoured by rock hyraxes. The area is characterized by an open steep landscape of basalt rocks, herbaceous vegetation cover and a sparse presence of *Ziziphus spina-christi* trees.

To evaluate the possible relationship between rock hyraxes and sand fly activity, field surveys were conducted during September 2017 on three occasions with intervals of four days between them. We observed rock-piles located along the dirt road during sunrise, using binoculars for detecting rock hyraxes from a distance of about 20–50 m (see trap locations in Fig. 1 and Additional file 1: Figure S2 as an example for the observations). Abundance levels of hyraxes were estimated in 16 randomly chosen rock-piles. Observations of rock hyraxes at a distance of < 50 m from the selected rock-piles were included in the counts. All observations were mapped using a GIS-GPS system (see Fig. 1). The elevation, slope and aspect of all points were roughly similar: east facing, 20–40 metres above sea level, and at a distance of 10–20 metres from the dirt road.

Sand fly traps were set on three occasions, on 3rd, 13th and 26th September 2017, in all 16 locations, on the third day of each of the observation sets before sunset, and were collected in the morning, immediately after the fourth survey day. Sand flies were counted, dissected and identified for all traps as described above.

The land cover at the observation points was evaluated using high-resolution true colour aerial photographs by using a DJI Phantom4 quadcopter at the time of the survey. Rock-piles and vegetation cover were delineated to polygons from the aerial photograph, in a 30 m buffer zone around each trapping point. Vegetation cover was evaluated for every identifiable living plant. All spatial processing was carried out using ArcMap 10.3.1 (ESRI 2015).

### The effects of rock hyraxes on the distribution of the sand flies

A two-step hierarchical analysis approach was used to assess the relationships between the sand fly and rock hyrax activity levels and the environmental variables, i.e. the size of the rock-piles and the extent of vegetation cover (trees or low vegetation). N-mixture analyses were applied to assess the sand fly activity levels, as was described above (data analysis), followed by linear regression analysis between the estimated values of the sand fly activity, the rock hyraxesʼ activities, and the land cover variables.

## Results

### The sand fly activity patterns in the land-use categories

**Fig. 3:**
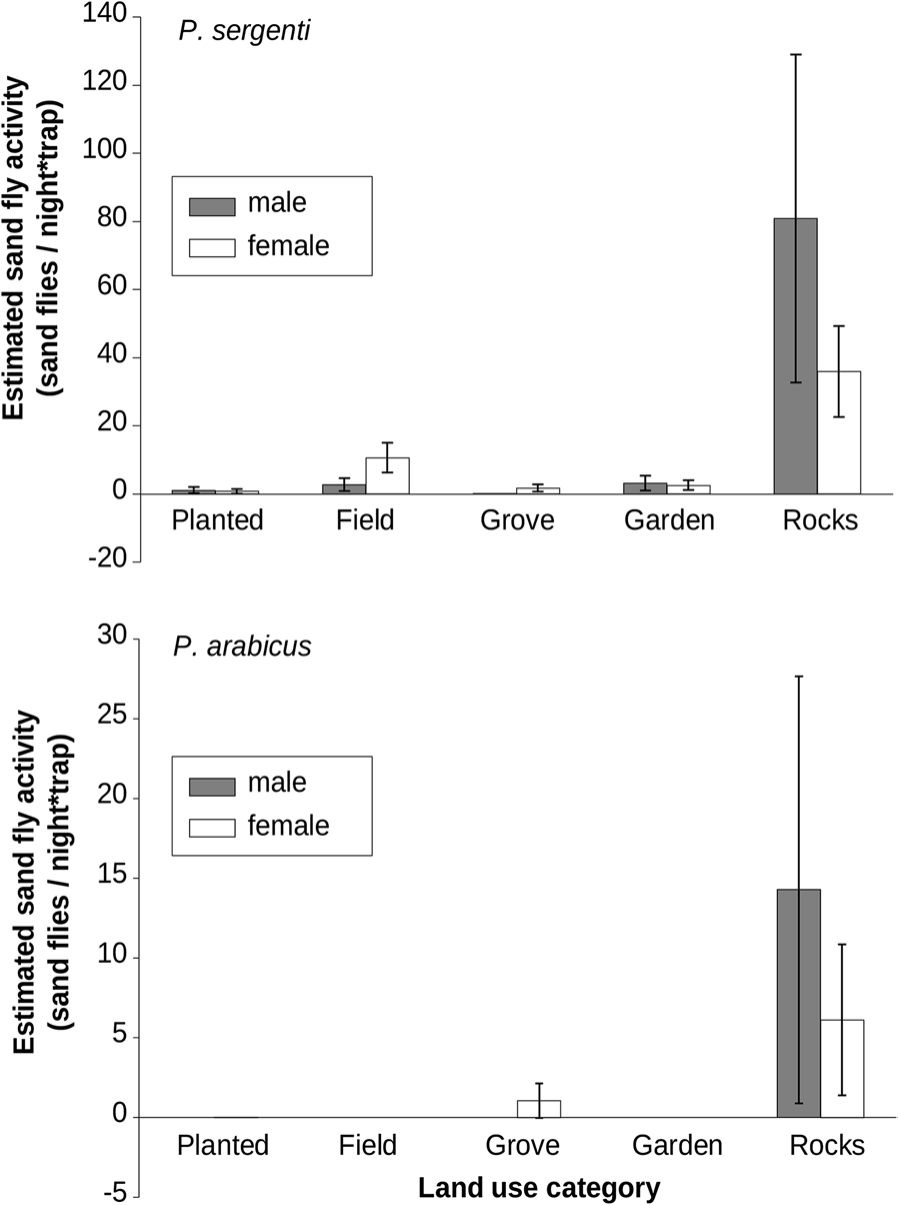
Sand fly activity within land use categories

The sand fly activity patterns of both species (*P. sergenti* and *P. arabicus*) and sexes were best fit by a negative binomial distribution; significant differences between land use types were detected (Table 1). The highest estimated values for both species and sexes were found at the rocks type. The estimated values of activity of *P. sergenti* were: males with 81 individuals (SD = 83.43), and females with 36 individuals (SD = 23.14). The lowest values were observed in the groves for males with 0 individuals, and for females in the planted areas with one individual (SD = 1.11), (Fig. 3). The highest estimated values of activity of *P. arabicus* were detected for both males, with 14 individuals (SD = 23.19), and females, with six individuals (SD = 8.17) at the rocks. The lowest values for males were found in all other four categories with 0 individuals, and for females in planted areas, as well as in fields and gardens with 0 individuals (Fig. 3). Captures for both species are detailed in Additional file 1: Table S1).

**Table 1:** Activity estimates by land use for *Phlebotomus* spp. The best-fit distribution type is noted

Species	Rank	Males		Females	
		Model	AIC	Model	AIC
*P. sergenti*	1	NB, time, LU	319.74	NB, LU	280.35
	2	NB, time	329.58	NB, time, LU	283.91
	3	NB, LU	381.68	NB	290.41
	4	NB	391.44	NB, time	293.97
*P. arabicus*	1	NB, time, LU	68.29	NB, time, LU	56.39
	2	NB, time	69.1	NB, time	57.1
	3	NB, LU	79.77	NB, LU	60.33
	4	NB	80.61	P, time, LU	60.47

*Notes*: “LU” denotes significant differences among land use types, and “time” denotes significant differences between sampling occasions

*Abbreviations*: P, Poisson, NB, negative binomial

### Movement patterns

**Fig. 4:**
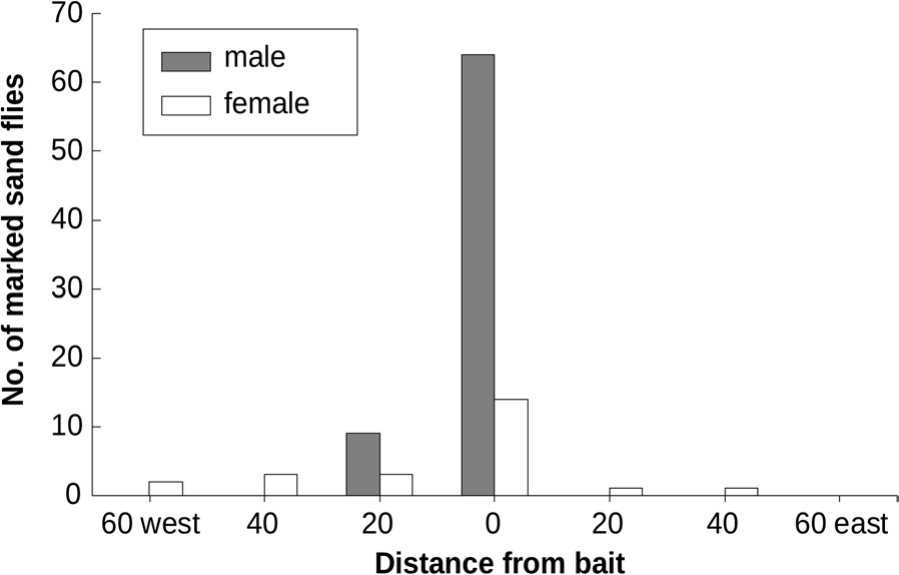
Marked *P. sergenti* at an interval distance of 20 metres from the sprayed bait in Elifelet

Marked *P. sergenti* individuals were captured in traps that were not located in rock-piles, and in the marked rocks. Only unmarked *P. arabicus* were caught in the traps located away from the rock-piles. The highest number of marked *P. sergenti* in a single trap was 20 for of males, and 6 for females, both in the same night of trapping (6th September, 2016), in the trap within the marked rocks. The highest number of males caught in traps away from the rocks was 7 at a distance of 20 m—on the west side of the marked rocks. The largest number of marked females away from the rocks was 3, 40 metres to the west. About 23 traps were found with no marked *P. sergenti.* Due to the low number of captured marked sand flies during each of the sessions, values were added together for each trap location for all five nights. The total number of flies, males and females, caught at the rock-pile exceeded 50 individuals, and exponentially declined with distance (Fig. 4). The numbers of individuals captured in the westward trap transect (towards the village) were much higher compared to the number of individuals caught in the eastward line transects but with no statistically significant differences. The model of exponential decrease with the distance best fit the data, with the lowest AIC value (61.24) (Table 2). Capture results are detailed in Additional file 1: Table S2.

**Table 2:** Model comparison for marked *P. sergenti* occurrence with distance from the coloured bait and directions

Model rank	Model	Parameter values	Log-likelihood	AIC
1	$\beta_{0} e^{{ - \beta_{1} Distance}}$	*β*_*0*_ = 59.4 *β*_*1*_ = 0.04	29.62	61.24
2	$\beta_{0} e^{{ - \left( {\beta_{1} DistEast + \beta_{2} DistWest} \right)}}$	*β*_*0*_ = 11.97 *β*_*1*_ = 0.65 *β*_*2*_ = 0.13	29.81	62.63
3	$\beta_{0} Dist$	*β*_*0*_ = 23.75	31.29	63.58
4	$\beta_{0} - \beta_{1} DistEast - \beta_{2} DistWest$	*β*_*0*_ = 11.97 *β*_*1*_ = 0.65 *β*_*2*_ = 0.7	30.81	64.62

Linear regression for sand flies vs distance did not reveal significant results (*R*^2^ = 0.53, *P* = 0.62). However, a natural-log transformation (ln x + 1) for the number of sand flies yielded a significant correlation (*R*^2^ = 0.63, *P* = 0.03).

### Host-sand fly relationships

An assessment of the sand fly activities using N-mixture models, based on three trapping occasions, yielded for both sexes of *P. sergenti* differences between observation sites and time. *Phlebotomus arabicus* activity, however, revealed differences between sites for both sexes but with no differences in time, i.e. uniform activity levels during the trapping nights. The activity levels of both species and sexes were best fitted with a Poisson distribution. The estimated activity of the rock hyraxes was evaluated from twelve surveys and showed differences between sites and time (Table 3). Captures are detailed in Additional file 1: Table S3.

**Table 3:** Activity estimates by site for *Phlebotomus.* spp. in the hilly Jordan river area. The best-fit distribution type is indicated

Species	Rank	Males		Females	
		Model	AIC	Model	AIC
*P. sergenti*	1	P, time, site	1146.58	P, time, site	542.38
	2	NB, time, site	1148.58	NB, time, site	544.38
	3	NB, time	1195.4	NB, time	581.09
	4	P, site	1237.2	P, site	684.24
*P. arabicus*	1	P, time, site	515.7	P, 1, site	487.31
	2	NB, time, 1	557.56	NB, 1, site	489.31
	3	P, 1, site	641.35	P, time, site	490.09
	4	NB, 1, 1	683.36	NB, 1, 1	517.33

*Notes*: “site” indicates significant differences among rock-piles, and “time” indicates significant differences among the sampling events

*Abbreviations*: P, Poisson, NB, negative binomial

**Fig. 5:**
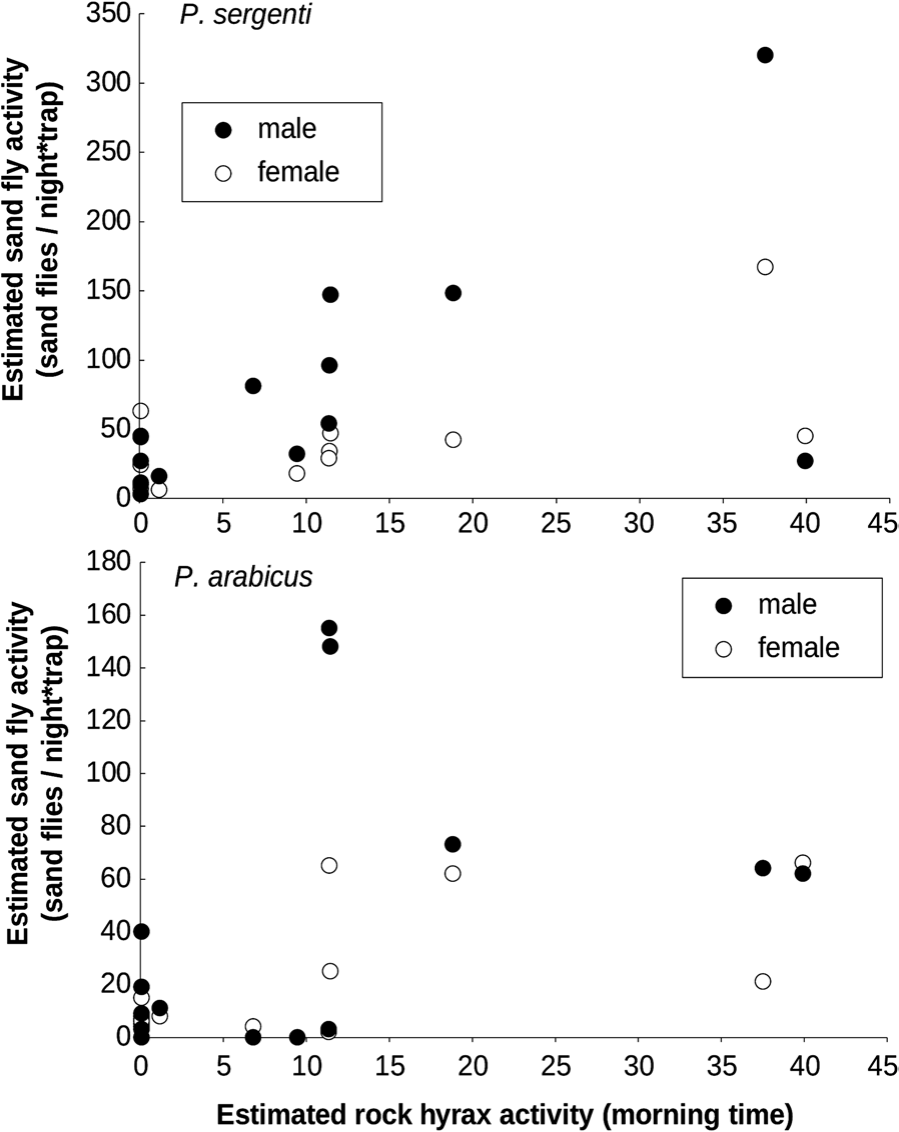
Linear regression between estimated sand fly activity (N-mixture models) and rock hyrax activity

**Fig. 6:**
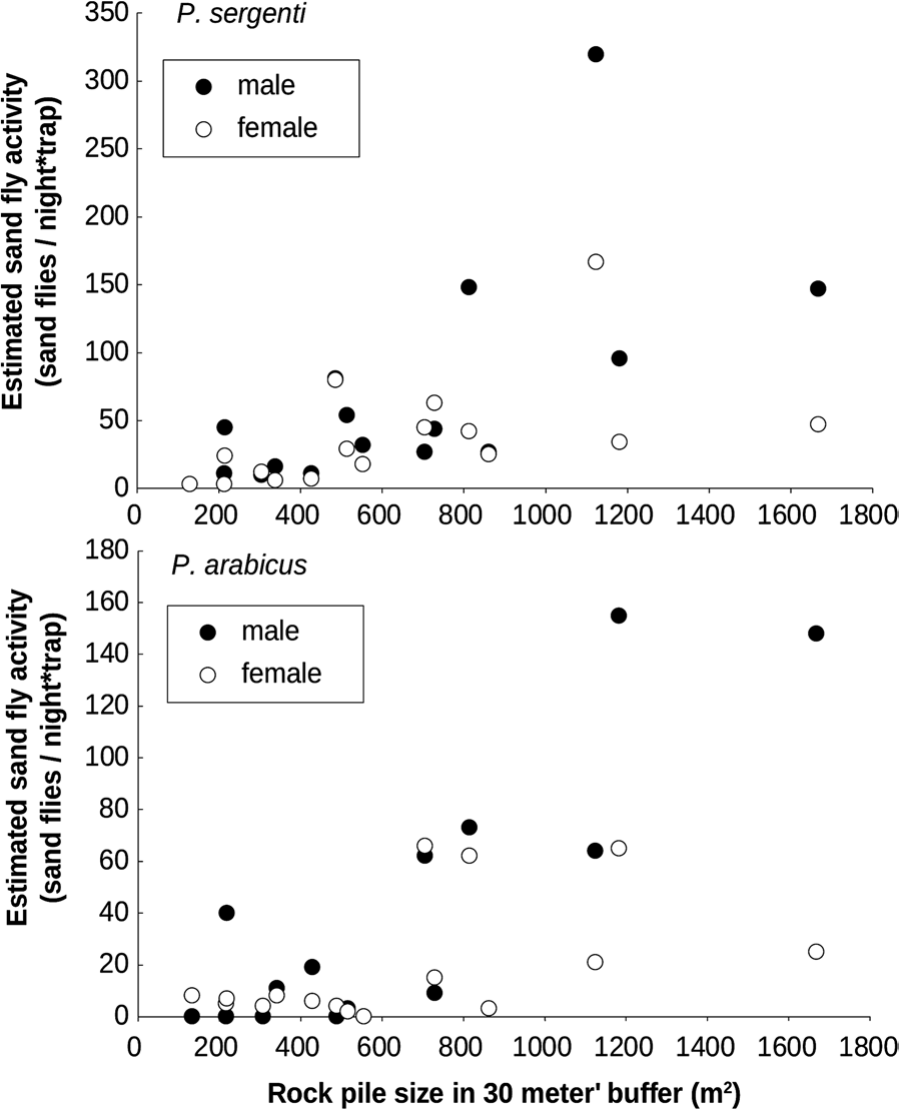
Linear regression between estimated sand fly activity (N-mixture models) and rock-pile size within a 30-metre buffer from trap location

*Phlebotomus sergenti* activity was significantly and positively correlated with rock-pile size and as well as with rock hyrax activity level. In contrast, the activity level of *P. arabicus* males was significantly correlated with rock-pile size (*R*^2^ = 0.62, *P* = 0.0002) and the activity level of females was significantly correlated with hyrax activity levels (*R*^2^ = 0.35, *P* = 0.008) (Figs. 5, 6 and Table 4). We further tested whether significant differences exist between the slopes (i.e. the response) of male and female *P. sergenti* to rock-pile size and hyrax activity. With respect to rock-pile size, slope values were slightly higher for males, but not significantly different from those of the females (*Z* = 1.53, *P* = 0.065) (Table 4). Male *P. sergenti* exhibited a stronger relationship with hyrax activity level, which was also not significantly different from the response of the females (*Z* = 1.38, *P* = 0.0838). Rock hyrax activity was not correlated with trees (*R*^2^ = 0.003, *P* = 0.85), low vegetation (*R*^2^ = 0.009, *P* = 0.73) or the size of the rock-piles (*R*^2^ = 0.22, *P* = 0.066).

**Table 4:** Regression analysis for sand fly activity with respect to hyrax activity and rock-pile size

Species	Covariate	Adj. *R*^2^	Slope	*P*-value
*P. sergenti*	Rock-pile size			
	Males	0.38	0.12	0.005
	Females	0.20	0.05	0.043
	Hyrax activity			
	Males	0.37	4.06	0.007
	Females	0.39	2.08	0.005
*P. arabicus*	Rock-pile size			
	Males	0.62	0.10	0.0002
	Females	0.18	0.02	0.0522
	Hyrax activity			
	Males	0.12	1.72	0.094
	Females	0.35	1.15	0.008

Notes: Adj. *R*^2,^ adjusted *R*^2^; slope, slope of the regression line; *P*-value, parameter significance value

Correlations between activity levels of males and females were positive for both species (*P. sergenti*: *R*^2^ = 0.77, *P* < 0.0001; *P. arabicus*: *R*^2^ = 0.53, *P* = 0.001), while there was only a low apparent correlation between species for males (*R*^2^ = 0.275, *P* = 0.037), or no correlation for females (*R*^2^ = 0.04, *P* = 0.47).

## Discussion

This study is focused on the activity and spatial distribution of the vectors of *L. tropica* (*P. sergenti* and *P. arabicus*) and on the host-sand fly relationship in rural and semi-natural Mediterranean landscapes, characterized by various types of land use. In all three monitoring experiments, the abundance of *P. sergenti* was greater than that of *P. arabicus*, as was also found in a previous study in the Korazim Heights [35]. Commonly, the trap results indicated significant differences between trap locations and time at Elifelet with a negative binomial distribution and in the hilly Jordan river area with a Poisson distribution. In addition, rock hyrax activity revealed a similar Poisson distribution with significant differences along space and time, which are beyond the scope of this research.

### Land use and sand fly activity

Our results demonstrate an almost exclusive preference for rocky habitats in comparison to other land-uses both for vectors and sexes. *Phlebotomus sergenti* activity in rocks was higher by an order of magnitude compared to other types of land use. This result conforms with those found in previous studies, which recognized natural and artificial rocky land-cover as the main habitats for *P. sergenti* [16, 30, 31]. A small number of *P. sergenti* females was found in other types of land use, mostly in open fields, which may indicate their higher mobility in seeking blood sources (see below *P. sergenti* movement patterns).

*Phlebotomus arabicus*, whose role as a vector for *L. tropica* is unique to the Korazim Heights in the Eastern Galilee [10], has been mentioned only in minor notes of early ecological studies. Kravchenko et al. [16] identified irrigated habitats, such as groves and gardens, as the preferred habitat of *P. arabicus* over rock-piles. In contrast, our results which contradict this study, provide a new observation of the exclusive preferences of *P. arabicus* for rocky habitats, and almost no activity in others.

Rock-piles seem to be a favoured habitat of sand flies as a result of several possible reasons: improved climate conditions (see Waitz el al. [35]), proximity to blood sources (mostly rock hyraxes), and protection from predation. This may lead to the assumption that both *L. tropica* vectors are strongly associated with rocky habitats due to a close relationship with the host, i.e. rock hyraxes.

### *Phlebotomus sergenti* movement patterns

Unlike *P. papatasi*, which was shown to exhibit high mobility properties [37], *P. sergenti* activity declined sharply away from the rocks; they tended to be active almost solely near their shelter, while only a slight activity was detected at a distance from the rocks. The range of movements was found to be marginally higher in females than in males, with a certain tendency to the western side, but with a weak statistical significance. The higher mobility of females due to their need for blood sources has been recognized in previous studies [35, 36]. However, our findings regarding their high presence near rocks may indicate the beneficial presence of rock hyraxes and other warm-blooded animals in the shelter of the rocks.

The higher prevalence of female individuals on the western side agrees with a previous study [32] from the Judean Desert in the town of Maʼale Adumim, where a higher activity of *P. sergenti* was found along the western side of the town (on the eastern-facing slope) which may be related to wind conditions. In Elifelet, the prevailing wind direction is from the west to the east, opposing the direction of the meal-seeking flight. During events of low wind conditions, which do not limit the sand fly activity, air may transport CO_2_ from the inhabited area of the village, and consequently, may attract female sand flies. However, this assumption requires further research.

### The relationships between hosts and sand flies

Both sand fly species exhibited a strong correlation between the sexes but only a poor correlation between the species, which indicates an absence of interspecific competition over resources. With the exception of *P. arabicus* males, both species showed a significant positive correlation with rock hyrax activity. This raises the possibility of the females being attracted to rock hyraxes as a dominant blood source which, in turn, attracts male individuals to the vicinity of rock hyrax populations for mating.

Male sand flies of both species exhibited higher activity levels in large rocky habitats and tended to aggregate within these habitats with a low correlation with rock hyrax presence in the case *P. arabicus*. The response of *P. sergenti* implies a slightly stronger effect of the rock-pile size and may explain the higher numbers of individuals, compared to *P. arabicus*. This complex spatial behaviour is possibly related to the low dispersal abilities of male sand flies, which would select a static strategy for activity in the preferred habitat without responding to the changes in rock hyrax activity in space.

These results lead to the insight that rock-piles are the dominant breeding and resting sites of both sand fly species which are mostly active during early night time (see Waitz et al. [35]). This aggregating behaviour of males may imply a Lek courting behaviour inside the rocky habitats that provide a protected site for breeding, close to a preferred blood source, i.e. the rock hyrax populations. Still, the prior preference of male individuals is targeted to large rock-piles, not necessarily close to rock hyraxes at the times of observation. As mentioned above, no correlation between sand fly activity levels and vegetation cover has been detected in the surveys, thus emphasizing the centrality of rocks as the most important habitat for *P. sergenti* and *P. arabicus* distribution.

The rapid growth of the human population in Israel over the last few decades and the establishment of new villages and neighbourhoods in the mountainous regions, have resulted in massive land use changes across the country. In many areas where the land is covered by rocks (such as basalt in the Eastern Galilee, or limestone and dolomite in most of the mountainous regions), they were cleared aside into rock-piles and replaced by different land use types such as agriculture, new towns and massive road construction. Soon, the new and widespread environment suitable for rock hyraxes became the dominant artificial habitat, replacing previous natural habitats such as rock crevices and caves [8]. Consequently, the extensive presence of rock hyraxes in the vicinity of many settlements and new towns increased the risk of the prevalence of *L. tropica* among human populations in the region [9].

## Conclusions

The present study demonstrates the links between rock-piles, rock hyrax and *P. sergenti*, as well as the consequent implications for human infection with *L. tropica.* The affinity of *P. sergenti* and *P. arabicus* for an exclusive environmental feature, i.e. rocky habitats, has been shown. These landscape features are also strongly associated with the presence of rock hyrax populations. The close association between the vector and the host of *L. tropica*, close to human populated areas, emphasized the role of rock-piles which mainly resulted from changes in land use by human activity. This crucial modification of the landscape, led to the increase of *L. tropica* occurrence.

## Additional file

**Additional file 1: Figure S1.** Illustration of traps located in the field, from distances of 0 meters to 60 meters, west-east, 5th October 2016. **Figure S2.** Example of an observation of rock hyraxes on a rock pile along the hilly Jordan area, 11th October 2017. **Table S1.** Elifelet sand fly captures: number caught in different land uses. **a** *P. sergenti.* **b** *P. arabicus. Abbreviations*: P, planted; F, field; G, grove; H, house; R, rocks. **Table S2.** Marked *P. sergenti* captures along the distance-transect traps: males + females. **Table S3.** Host-sand fly relationships - Hilly Jordan sand fly captures. **a** *P. sergenti*. **b** *P*. *arabicus.*

## Data Availability

Data supporting the conclusions of this article are included within the article and its additional files. The datasets used and analyzed for the study are available from the authors upon a reasonable request.
