# Supplementary material for: Effects of land use type, spatial patterns and host presence on Leishmania tropica vectors activity
Source: Parasit Vectors. 2019 Jun 25;12:320. doi: 10.1186/s13071-019-3562-0 (PMC6593564; doi:10.1186/s13071-019-3562-0)
Supplement: Supplementary file 1 — Additional file 1: Figure S1. Illustration of traps located in the field, from distances of 0 meters to 60 meters, west-east, 5th October 2016. Figure S2. Example of an observation of rock hyraxes on a rock pile along the hilly Jordan area, 11th October 2017. Table S1. Elifelet sand fly captures: number caught in different land uses. a P. sergenti. b P. arabicus. Abbreviations: P, planted; F, field; G, grove; H, house; R, rocks. Table S2. Marked P. sergenti captures along the distance-transect traps: males + females. Table S3. Host-sand fly relationships - Hilly Jordan sand fly captures. a P. sergenti. b P. arabicus. [file 13071_2019_3562_MOESM1_ESM.docx]

**Additional file 1: Figure S1.** Illustration of traps located in the field, from distances of 0 meters to 60 meters, west-east, 5^th^ October 2016.


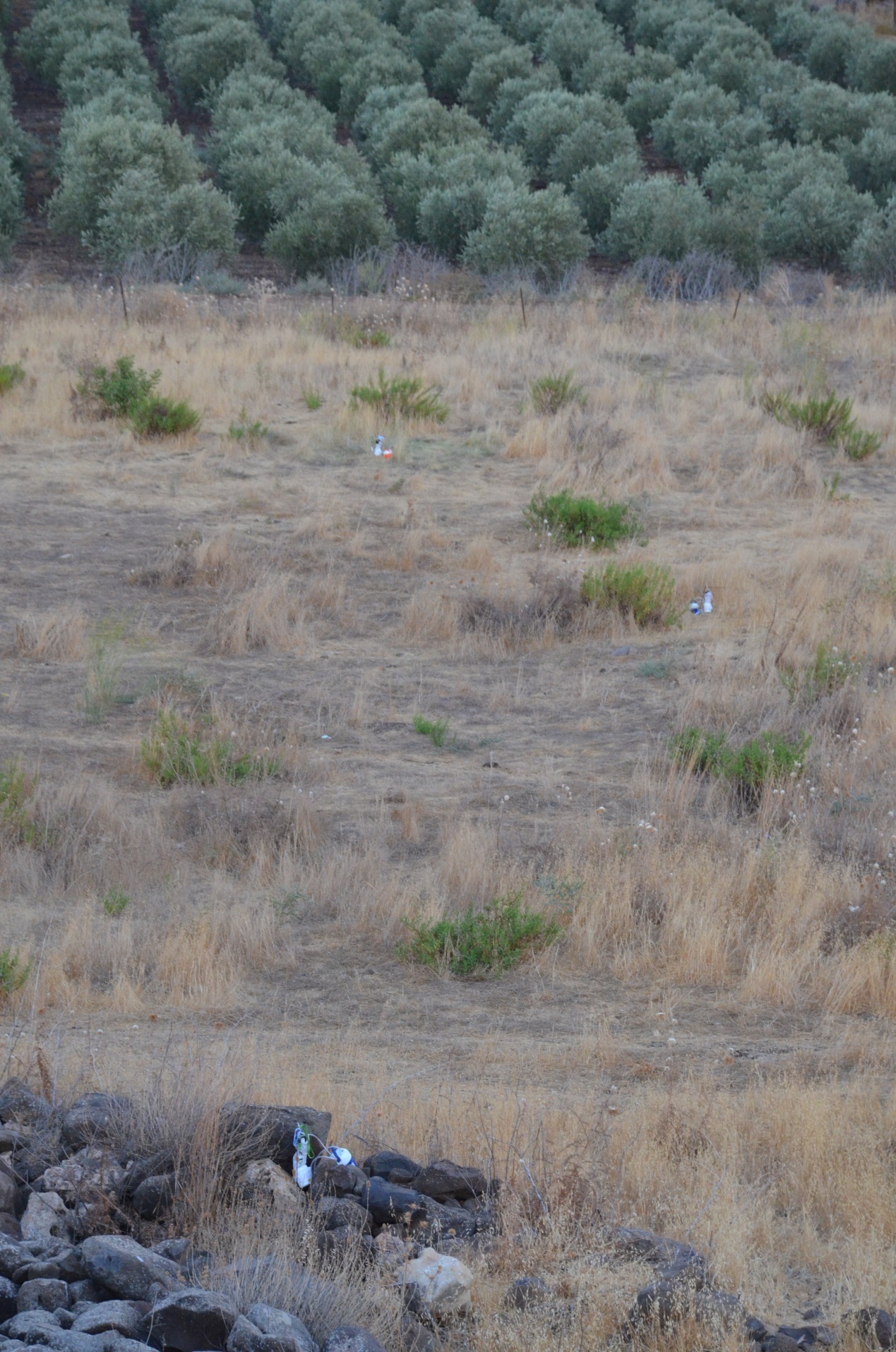


**Additional file 1: Figure S2.** Example of an observation of rock hyraxes on a rock pile along the hilly Jordan area. 11^th^ October 2017.


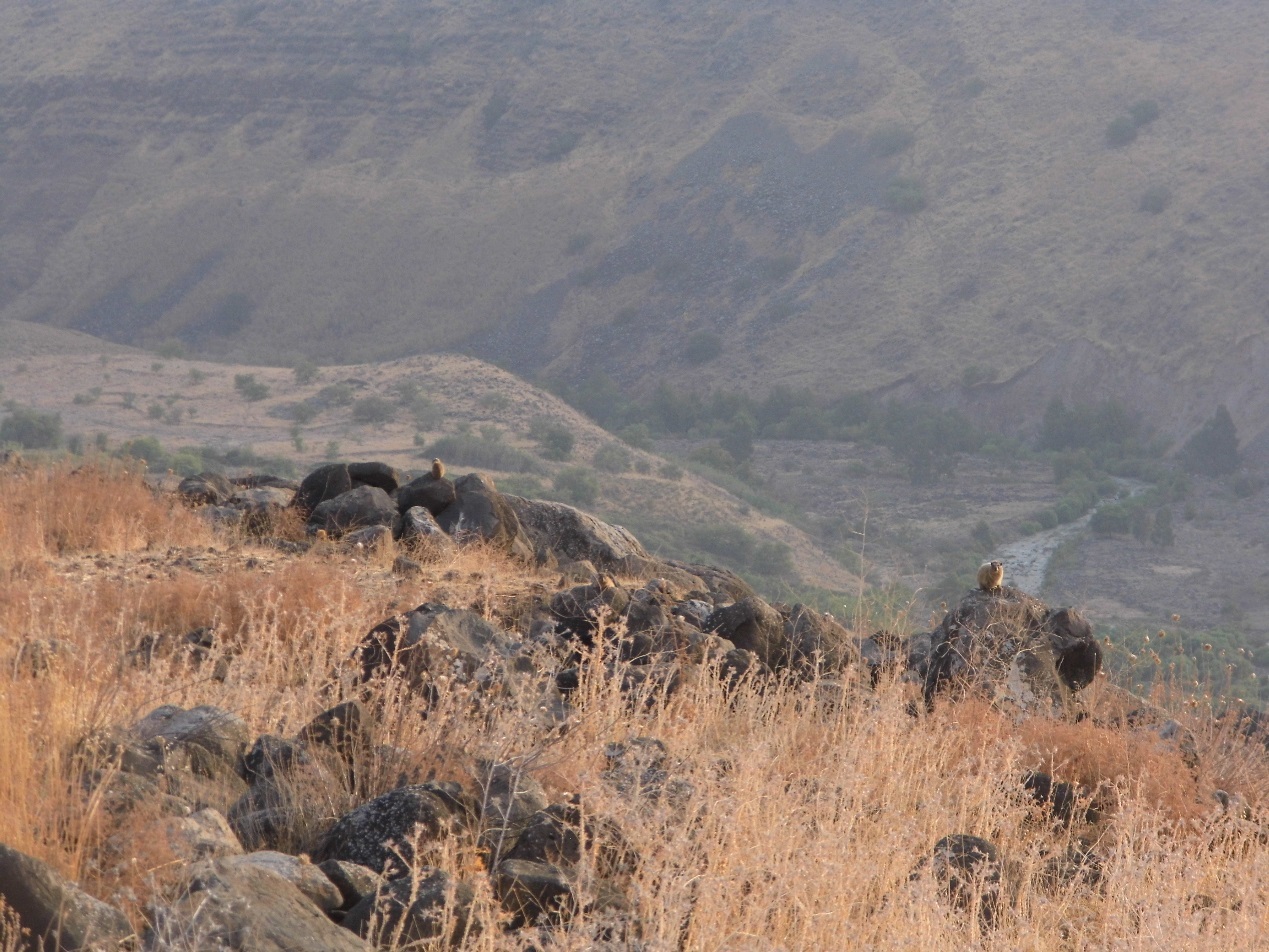


**Additional file 1: Table S1.** Elifelet sand flies' captures: number caught in different land uses. P = Planted,

F = Field, G =Grove, H = House, R = Rocks.

*a. P. sergenti*

| Date | 11^th^ July 2016 | | 31^st^ July 2016 | | 24^th^ August 2016 | |
| --- | --- | --- | --- | --- | --- | --- |
| Trap | Male | Female | Male | Female | Male | Female |
| p1 | 1 | 0 | 0 | 0 | 0 | 0 |
| p2 | 0 | 0 | 0 | 0 | 0 | 0 |
| p3 | 0 | 0 | 0 | 0 | 2 | 2 |
| f1 | 0 | 7 | 0 | 0 | 0 | 0 |
| f2 | 7 | 4 | 3 | 1 | 0 | 12 |
| f3 | 0 | 6 | 0 | 0 | 0 | 0 |
| g1 | 0 | 2 | 0 | 0 | 0 | 1 |
| g2 | 0 | 0 | 0 | 0 | 0 | 1 |
| g3 | 0 | 0 | 0 | 1 | 0 | 0 |
| h1 | 0 | 0 | 0 | 0 | 0 | 0 |
| h2 | 3 | 4 | 1 | 3 | 7 | 4 |
| h3 | 1 | 0 | 1 | 0 | 0 | 0 |
| r1 | 58 | 23 | 28 | 6 | 104 | 16 |
| r2 | 26 | 17 | 90 | 52 | 61 | 28 |
| r3 | 3 | 2 | 4 | 2 | 15 | 6 |

b. *P. arabicus*

| Date | 11^th^ July 2016 | | 31^st^ July 2016 | | 24^th^ August 2016 | |
| --- | --- | --- | --- | --- | --- | --- |
| Trap | Male | Female | Male | Female | Male | Female |
| p1 | 0 | 0 | 0 | 0 | 0 | 0 |
| p2 | 0 | 0 | 0 | 0 | 0 | 0 |
| p3 | 0 | 0 | 0 | 0 | 0 | 0 |
| f1 | 0 | 0 | 0 | 0 | 0 | 0 |
| f2 | 0 | 0 | 0 | 0 | 0 | 0 |
| f3 | 0 | 0 | 0 | 0 | 0 | 0 |
| g1 | 0 | 2 | 0 | 0 | 0 | 0 |
| g2 | 0 | 0 | 0 | 0 | 0 | 0 |
| g3 | 0 | 0 | 0 | 0 | 0 | 0 |
| h1 | 0 | 0 | 0 | 0 | 0 | 0 |
| h2 | 0 | 0 | 0 | 0 | 0 | 0 |
| h3 | 0 | 0 | 0 | 0 | 0 | 0 |
| r1 | 18 | 8 | 9 | 3 | 28 | 1 |
| r2 | 0 | 0 | 6 | 2 | 4 | 1 |
| r3 | 0 | 0 | 0 | 0 | 0 | 0 |

**Additional file 1: Table S2.** Marked *P. sergenti* captures along the distance-transect traps: males + females

| Direction | West | | | Rock pile | East | | |
| --- | --- | --- | --- | --- | --- | --- | --- |
| Date/Distance (m') | 60 | 40 | 20 | 0 | 20 | 40 | 60 |
| 3^rd^ August 2016 | 1 | 0 | 0 | 11 | 1 | 0 | 0 |
| 6^th^ September 2016 | 1 | 0 | 1 | 26 | 0 | 0 | 0 |
| 11^th^ September 2016 | 0 | 3 | 1 | 17 | 0 | 0 | 0 |
| 20^th^ September 2016 | 0 | 0 | 8 | 10 | 0 | 1 | 0 |
| 5^th^ October 2016 | 0 | 0 | 2 | 14 | 0 | 0 | 0 |
| Total | 2 | 3 | 12 | 78 | 1 | 1 | 0 |

**Additional file 1: Table S3.** Host-sand flies' relationships - Hilly Jordan sand flies' captures.

a. *P. sergenti*

| Date | 4^th^ September 2017 | | 13^th^ September 2017 | | 27^th^ September 2017 | |
| --- | --- | --- | --- | --- | --- | --- |
| Trap | Male | Female | Male | Female | Male | Female |
| 1 | 51 | 66 | 16 | 12 | 58 | 43 |
| 2 | 23 | 14 | 6 | 11 | 21 | 6 |
| 3 | 9 | 3 | 11 | 5 | 6 | 0 |
| 4 | 209 | 157 | 150 | 98 | 310 | 80 |
| 5 | 52 | 35 | 123 | 15 | 33 | 16 |
| 6 | 20 | 39 | 17 | 21 | 5 | 5 |
| 7 | 123 | 25 | 28 | 9 | 53 | 39 |
| 8 | 9 | 11 | 62 | 15 | 70 | 28 |
| 9 | 6 | 8 | 6 | 8 | 6 | 6 |
| 10 | 15 | 17 | 7 | 6 | 20 | 19 |
| 11 | 3 | 3 | - | - | 9 | 0 |
| 12 | 24 | 19 | 30 | 17 | - | - |
| 13 | 31 | 38 | 6 | 14 | 30 | 49 |
| 14 | 14 | 14 | 1 | 10 | 39 | 17 |
| 15 | 0 | 0 | 3 | 3 | 0 | 0 |
| 16 | 3 | 6 | 4 | 1 | 9 | 4 |

b. *P*. *arabicus*

| Date | 4^th^ September 2017 | | 13^th^ September 2017 | | 27^th^ September 2017 | |
| --- | --- | --- | --- | --- | --- | --- |
| Trap | Male | Female | Male | Female | Male | Female |
| 1 | 0 | 3 | 0 | 0 | 0 | 0 |
| 2 | 0 | 0 | 0 | 0 | 0 | 0 |
| 3 | 9 | 0 | 0 | 5 | 0 | 4 |
| 4 | 30 | 13 | 7 | 3 | 37 | 8 |
| 5 | 37 | 20 | 35 | 44 | 16 | 0 |
| 6 | 33 | 50 | 22 | 42 | 21 | 14 |
| 7 | 71 | 7 | 46 | 7 | 71 | 15 |
| 8 | 34 | 12 | 13 | 7 | 145 | 56 |
| 9 | 0 | 0 | 0 | 1 | 0 | 3 |
| 10 | 0 | 1 | 2 | 1 | 0 | 2 |
| 11 | 0 | 0 | - | - | 0 | 3 |
| 12 | 1 | 0 | 1 | 1 | - | - |
| 13 | 7 | 4 | 0 | 2 | 0 | 10 |
| 14 | 12 | 2 | 1 | 2 | 28 | 4 |
| 15 | 0 | 3 | 0 | 0 | 0 | 5 |
| 16 | 11 | 1 | 8 | 2 | 4 | 4 |
